# Salivary miR-21 as a Lifestyle-Responsive Biomarker: A Critical Appraisal and Future Perspectives

**DOI:** 10.31557/APJCP.2026.27.2.399

**Published:** 2026-02-05

**Authors:** Nathkapach Kaewpitoon Rattanapitoon, Schawanya Kaewpitoon Rattanapitoon

**Affiliations:** 1 *FMC Medical Center, Nakhonratchasima, Thailand.*; 2 *Parasitic Disease Research Center, Nakhonratchasima, Thailand. *

**Dear Editor**

We read with interest the recent publication by Fadhil et al., entitled “Evaluation of Salivary Carcinogenic microR-21 and miR-125a Expression Associated with Alcohol Consumption and Smoking”, published in the Asian Pacific Journal of Cancer Prevention [1]. The study addresses an important and timely topic at the intersection of molecular epidemiology and lifestyle-related cancer risk, and contributes to the growing body of research on non-invasive biomarkers.

The authors’ selection of miR-21 as a molecular target is well justified. MiR-21 has been extensively reported as an oncogenic microRNA implicated in a wide range of malignancies, including lung, breast, colorectal, and melanoma cancers [2, 3]. In this context, the observation of elevated salivary miR-21 expression among individuals with smoking and alcohol consumption habits provides additional evidence supporting the association between lifestyle-related exposures and miRNA deregulation. While the current findings are derived from a cross-sectional analysis, they suggest that miR-21 may reflect molecular changes associated with carcinogenic exposures. The inclusion of miR-125a as a comparative miRNA further strengthens the study design. The lack of significant alteration in miR-125a expression is consistent with reports indicating differential regulatory behavior among salivary miRNAs in response to environmental and lifestyle factors [4].

The use of saliva as the biological matrix represents a notable methodological strength. Saliva collection is non-invasive, cost-effective, and feasible for large-scale population studies. Previous investigations have demonstrated that salivary miRNAs can remain stable due to protective mechanisms such as encapsulation within extracellular vesicles, supporting their suitability for biomarker research [5]. In this regard, the authors’ work contributes to the practical advancement of salivary miRNA analysis in lifestyle-associated cancer research.

Nevertheless, several aspects merit consideration in future studies. First, although the sample size employed is appropriate for an exploratory investigation, larger cohorts across multiple centers would enhance statistical power and external validity. Expanded sample sizes would also permit stratified analyses by demographic variables, including age, sex, and socioeconomic status, which have been reported to influence salivary miRNA expression patterns [6].

Second, the categorical classification of smoking and alcohol consumption could be complemented by more detailed exposure assessments. Quantitative measures, such as pack-years of smoking or estimated alcohol intake, may provide deeper insight into exposure-associated trends in miRNA expression. Prior studies have indicated that varying levels of lifestyle exposure are associated with differential salivary miRNA profiles, supporting the value of such an approach [7].

Third, longitudinal study designs would be valuable to clarify temporal relationships between lifestyle exposures and miR-21 expression. Cross-sectional analyses cannot determine whether observed miRNA alterations precede or follow exposure-related biological changes. Longitudinal data may help to assess whether changes in lifestyle behaviors are accompanied by corresponding modulation of salivary miRNA levels, thereby strengthening the evidence for their use as dynamic biomarkers [8].

From a mechanistic perspective, further investigation into the molecular pathways linking smoking and alcohol exposure to miR-21 upregulation is warranted. Existing evidence suggests that inflammatory and oxidative stress–related signaling pathways, including NF-κB and STAT3, may contribute to miR-21 induction under conditions of chronic exposure [9]. Further elucidation of these mechanisms may help provide additional biological context for the observed associations.

Finally, while the current study focuses on two miRNAs, future research may benefit from incorporating broader salivary miRNA panels. Other salivary miRNAs have been implicated in carcinogenic exposure and oral pathology, and multiplex profiling approaches may improve diagnostic performance and risk stratification [10].

In conclusion, the study by Fadhil et al. provides valuable preliminary evidence supporting an association between salivary miR-21 expression and lifestyle-related exposures. The findings contribute meaningfully to ongoing efforts aimed at identifying non-invasive molecular indicators relevant to cancer risk assessment. Further studies with expanded cohorts, refined exposure assessment, and longitudinal follow-up will be important to clarify the clinical and preventive implications of salivary miR-21.

## References

[1] Fadhil R, Good D, Wei MQ (2025). Evaluation of salivary carcinogenic microR-21 and miR-125a expression associated with alcohol consumption and smoking. Asian Pac J Cancer Prev..

[2] Hashemi M, Mirdamadi MSA, Talebi Y, Khaniabad N, Banaei G, Daneii P (2023). Pre-clinical and clinical importance of miR-21 in human cancers: Tumorigenesis, therapy response, delivery approaches and targeting agents. Pharmacol Res..

[3] Feng YH, Tsao CJ (2016). Emerging role of microRNA-21 in cancer. Biomed Rep..

[4] Mead EA, Boulghassoul-Pietrzykowska N, Wang Y, Anees O, Kinstlinger NS, Lee M (2022). Non-invasive microRNA profiling in saliva can serve as a biomarker of alcohol exposure and its effects in humans. Front Genet..

[5] Vageli DP, Doukas PG, Shah R, Boyi T, Liu C, Judson BL (2023). A novel saliva and serum miRNA panel as a potential useful index for oral cancer and the association of miR-21 with smoking history: a pilot study. Cancer Prev Res..

[6] Al Rawi N, Elmabrouk N, Abu Kou R, Mkadmi S, Rizvi Z, Hamdoon Z (2021). The role of differentially expressed salivary microRNA in oral squamous cell carcinoma. A systematic review. Arch Oral Biol..

[7] Öngöz Dede F, Gökmenoğlu C, Türkmen E, Bozkurt Doğan Ş, Ayhan BS, Yildirim K (2023). Six miRNA expressions in the saliva of smokers and non-smokers with periodontal disease. J Periodontal Res..

[8] Zahran F, Ghalwash D, Shaker O, Al-Johani K, Scully C (2015). Salivary microRNAs in oral cancer. Oral Dis..

[9] Sheedy FJ (2015). Turning 21: Induction of miR-21 as a key switch in the inflammatory response. Front Immunol..

[10] Momen-Heravi F, Trachtenberg AJ, Kuo WP, Cheng YS (2014). Genomewide study of salivary microRNAs for detection of oral cancer. J Dent Res..

