# Supplementary material for: Salivary miR-21 as a Lifestyle-Responsive Biomarker: A Critical Appraisal and Future Perspectives
Source: Asian Pac J Cancer Prev. 2026 Feb 5;27(2):399–400. doi: 10.31557/APJCP.2026.27.2.399 (PMC13499980; doi:10.31557/APJCP.2026.27.2.399)
Supplement: Related texts

# *Reply to the letter to the editor: *Salivary miR-21 as a Lifestyle-Responsive Biomarker: A Critical Appraisal and Future Perspectives

**Published:** 2026

**Dear Editor**

We sincerely appreciate the thoughtful and encouraging comments provided by [ Nathkapach and Schawanya] regarding our article, “Evaluation of Salivary Carcinogenic microR-21 and miR-125a Expression Associated with Alcohol Consumption and Smoking.” We are pleased that our work has been recognized for its contribution to the field of salivary biomarker research and for highlighting the potential of miR-21 as an early indicator of carcinogenic exposure. We fully agree with the suggestions raised concerning the need for larger, multicenter, and longitudinal studies, as well as precise exposure assessments to strengthen causal inferences. These valuable insights align well with our future research direction, which includes expanding the cohort size and exploring additional salivary miRNAs involved in lifestyle-related carcinogenesis. We thank the author(s) once again for their constructive feedback and interest in our study. Such scholarly engagement helps advance the shared goal of developing reliable, non-invasive biomarkers for cancer risk assessment and prevention.
